# Natural history of pain associated with melanoma surgery

**DOI:** 10.1097/PR9.0000000000000689

**Published:** 2018-10-19

**Authors:** Charlotte Slagelse, Troels Munch, Clara Glazer, Kaitlin Greene, Nanna Brix Finnerup, Mohammed Kashani-Sabet, Stanley P. Leong, Karin Lottrup Petersen, Michael C. Rowbotham

**Affiliations:** a California Pacific Medical Center Research Institute, San Francisco, CA, USA; b Department of Epidemiology, Aarhus University Hospital, Aarhus, Denmark; c Department of Clinical Medicine, The Danish Pain Research Center, Aarhus University, Aarhus, Denmark

**Keywords:** Melanoma, Sentinel node biopsy, Complete lymph node dissection, Neuropathic pain, Postoperative pain, Cancer pain, Sensory symptoms

## Abstract

**Introduction:**

After excision of a primary malignant melanoma (MM), treatment of stage IB or higher MM consists of sentinel lymph node biopsy (SLNB). If malignant cells are identified, a complete lymph node dissection (CLND) can be performed.

**Objective:**

To determine the natural history of pain and sensory changes after MM surgery.

**Methods:**

We prospectively followed 39 patients (29 SLNB-only, 2 CLND-only, and 8 CLND preceded by SLNB) from before inguinal or axillary surgery through 6 months after surgery on measures of pain intensity, sensory symptoms, allodynia, and questionnaires of anxiety, depression, and catastrophizing.

**Results:**

No patient had pain preoperatively. Ten days after surgery, 35% had surgical site pain after SLNB-only compared with 90% after CLND (*P* < 0.003); clinically meaningful pain (Visual Analogue Scale ≥ 30 mm/100 mm) was reported by 3% of patients after SLNB-only compared with 40% after CLND (*P* < 0.001). At 6 months, all SLNB-only patients were pain-free. By contrast, 4 of 7 in the SLNB + CLND group still had pain (*P* < 0.002). At 6 months, symptoms of altered sensation or numbness were reported by 32% and 42% of SLNB-only patients, and by 67% and 67% of patients undergoing CLND surgery (both *P* > 0.05).

**Conclusion:**

Acute pain is more common after CLND surgery. Undergoing SLNB followed by more invasive CLND surgery may increase the likelihood of pain at 6 months. Persistent sensory symptoms typical of those associated with nerve injury are more common after CLND. Surgery for MM is a good model for studying the natural history of postsurgical pain and sensory changes.

## 1. Introduction

Malignant melanoma (MM) is the fifth most common cancer in the United States with a projected estimate of 91,270 new cases and 9320 deaths in 2018.^1,16^ Treatment begins with local resection of the skin lesion for diagnosis and initial staging per American Joint Committee on Cancer (AJCC) criteria.^2^ If the melanoma stage is T1b (Breslow thickness ≥1 mm, presence of tumor ulceration) or higher, wide excision to ensure clear margins will be combined with a sentinel lymph node biopsy (SLNB) to determine whether cancer cells have metastasized. If melanoma cells are found in the sentinel node, all regional lymph nodes may be removed at a later date through complete lymph node dissection (CLND). The most common complications after SLNB and CLND include pain, sensory changes, seroma, lymphedema, and wound infection.^3,7,15,17,18^

In all body regions, the SLNB procedure typically requires a 3-cm incision to remove an average of 3 sentinel lymph nodes. For comparison, an axillary CLND incision is usually 7 to 10 cm long and an average of 15 to 20 lymph nodes is removed. The surgical field may expose the intercostobrachial, thoracodorsal, and long thoracic nerves. For an inguinal CLND, the incision is typically 15 to 20 cm long and an average of 10 to 15 lymph nodes is removed. The surgical field may expose the femoral, ilioinguinal, genitofemoral, and iliohypogastric nerves. Damage to these large nerves or smaller nerves in the surgical field can lead to sensory abnormalities and chronic neuropathic pain. Given the extent of tissue resection, reported complication rates of 50% from CLND have been reported.^17^ For melanoma, 2 retrospective studies reported sensory changes after MM surgery.^8,9^ Høimyr et al.^9^ reported 32% of 124 patients undergoing SLNB reported sensory changes and 14% reported pain at 2 years after surgery; of the 51 patients undergoing CLND, 84% reported sensory changes and 34% reported pain. Three prospective studies of pain after SLNB or CLND reported pain after surgery for MM (0.9%–8.9% of the patients). The study populations had similar demographics but were very heterogeneous in terms of length of follow-up, and their description of the definition of pain was lacking or minimal.^12,17,18^ A more extensive prospective literature exists within the field of breast cancer. However, breast cancer treatment often includes procedures that independently may contribute to postoperative pain, such as local radiotherapy and chemotherapy after lumpectomy or mastectomy, or neoadjuvant therapy consisting of a biopsy followed by chemotherapy and radiation before lumpectomy.

The purpose of this study was to prospectively describe the natural history of pain and sensory changes in MM patients undergoing only SLNB, only CLND, or SLNB followed by CLND.

## 2. Methods

### 2.1. Patients

At the Center for Melanoma Research and Treatment at California Pacific Medical Center (CPMC), all patients with newly diagnosed cutaneous MM scheduled to undergo axillary or inguinal SLNB or CLND at the CPMC between March and December of 2012 were invited to participate. Patients were diagnosed by a pathologist on the basis of a local resection of the skin lesion. Staging was performed according to AJCC criteria.^6^ To be eligible, patients had to have: an expected survival of more than 6 months, be able to speak English, and be willing to provide written informed consent. Exclusion criteria were neurological disorders, preexisting neuropathic pain, medication use that could interfere with pain ratings or sensory assessments, or previous surgery in the affected area. Eligibility was confirmed by medical history, review of medical records, and neurological examination. The study was approved by the CPMC Institutional Review Board and conducted in accordance with good clinical practice and the Helsinki declaration. All patients gave written consent before study inclusion.

### 2.2. Design

**Figure 1.**
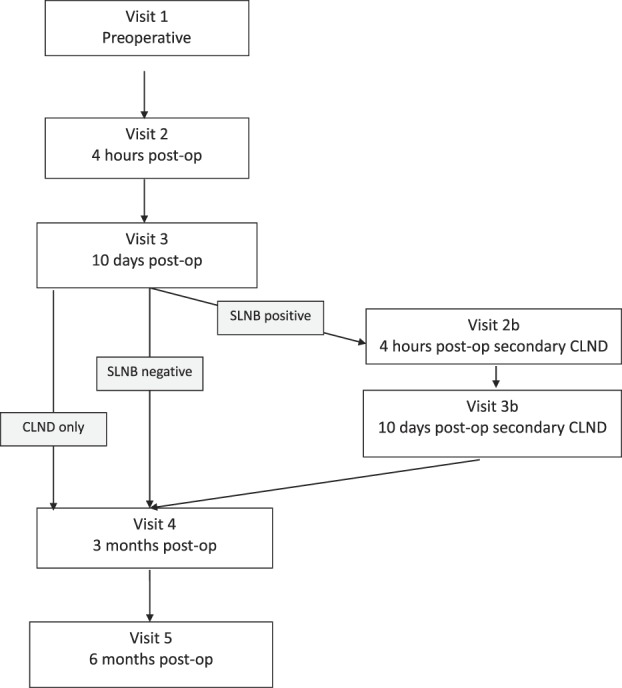
Study timeline. CLND, complete lymph node dissection; SLNB, sentinel lymph node biopsy.

The study was designed as a 5- to 7-session prospective observational study (Fig. 1). This design adheres to Kehlet and Rathmell's^11^ recommendations for studies of postoperative pain. Patients were examined preoperatively (V1, baseline visit, and usually within 5 days before surgery) and postsurgically (V2 after 4 hours, V3 after 10 days, V4 after 3 months, and V5 after 6 months). If any of the lymph nodes removed during SLNB contained malignant cells, the patient would return for CLND approximately 30 days later. These patients would then complete 2 additional postsurgical visits analogous to V2 and V3 after SLNB: (V2b) at 4 hours after CLND and (V3b) at 10 days after CLND. The 3-month and 6-month follow-up visits would then follow the date of the CLND surgery. Patients not able to attend in person for the V4 and V5 visits were contacted by phone to complete the interview and questionnaires, but physical examination and sensory evaluation could not be completed.

### 2.3. Surgical and anesthetic procedures

Surgical procedures were performed by S.P. Leong. Adhering to AJCC-staging and guidelines, general anesthesia with intravenous (i.v.) propofol combined with the nondepolarizing neuromuscular blocker rocuronium was used.^6^ Anesthesia was maintained with inhaled desflurane or sevoflurane. Analgesia was provided using i.v. fentanyl or i.v. hydromorphone during the surgery. In all patients, a local anesthetic (lidocaine 1% or Marcaine 0.25%) was injected around the site of excision and wound closure. Melanomas stage T1b or above more severe stages were all reexcised with 1- or 2-cm margins, with or without a skin graft, in combination with an SLNB.

The lymphatic drainage system was imaged preoperatively by lymphoscintigraphy using a Lymphoseek.^13^ Intraoperatively, a gamma-probe was used to identify possible sentinel lymph nodes (usually 1–3), which were then excised. The same technique was used in both inguinal and axillary regions. For patients with a positive SLNB, the CLND was performed within approximately thirty days. Complete lymph node dissection of the axilla was performed as en bloc resection of the lymphatic and fatty tissue of levels I, II, and III. The CLND of the groin was performed through an incision crossing the inguinal crease and en bloc resection of the contents of trigonum femorale.

### 2.4. Outcome measures

The schedule of events is shown in Table 1.

**Table 1:**
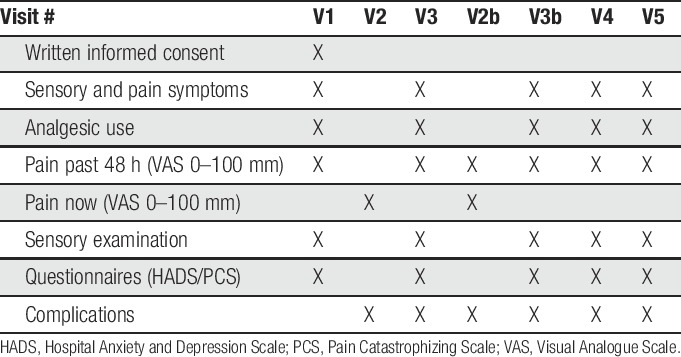
Schedule of events.

#### 2.4.1. Pain and analgesic use

Pain was defined as the presence of any pain using a 100-mm Visual Analogue Scale (VAS) anchored by 0 = “no pain” and 100 = “worst pain imaginable.” Clinically meaningful pain was defined as pain of intensity ≥30/100. The primary outcome measure, overall pain during the past 48 hours, was rated before any testing was performed. For visits V2 and V2b-only, because they occurred 4 hours after surgery, overall pain during the past 48 hours was not relevant and patients were only asked about current pain severity at the SLNB or CLND site. At all visits, patients were asked about use of either opioid or nonsteroidal anti-inflammatory drug analgesics for symptoms related to SLNB or CLND.

#### 2.4.2. Sensory and pain symptoms

Patients reported “yes/no” to the presence of 6 sensory symptoms (altered skin sensation, increased skin sensitivity, numbness, tingling, pins and needles, and itching) and 3 pain descriptors (burning, painful cold, and electrical shocks) at the surgical site (SLNB or CLND).

#### 2.4.3. Allodynia to brush stimuli

Allodynia severity was rated at all visits in the surgical sites (reexcision and SLNB or CLND) by gently stroking the skin with the 2.5-cm wide foam paintbrush 3 times at 1-second intervals. Patients and the investigator rated allodynia as present or absent. Allodynia ratings were not performed at V2 and V2b visits due to bandages and possible lingering effects of surgical local anesthesia.

#### 2.4.4. Von Frey hair testing

Sensitivity to mechanical stimulation was determined around the surgical sites (SLNB and/or CLND) using a 26.0-g von Frey hair. Each site was stimulated 3 times with 1 second between stimulations. After the 3 stimulations, patients were first asked whether they felt the stimulations (yes/no). If the stimulations were felt, the patients were asked whether stimulations were painful (yes/no) in comparison with a similar stimulus in an unaffected area.

#### 2.4.5. Questionnaires

The Hospital Anxiety and Depression Scale (HADS) is a 14-item questionnaire with measures of anxiety and depression.^19^ Each question is scored on a 0- to 3-point Likert scale allowing for a maximum of 21 points on each scale. For each scale, a score <8 is considered normal, a score of 8 to 11 is considered borderline abnormal, and a score >11 is considered abnormal.

The Pain Catastrophizing Scale (PCS) is a patient-completed, 13-item questionnaire.^16^ Each item is scored on a 0- to 4-point Likert scale for a maximum of 52 points. The PCS is designed to tap into 3 dimensions of catastrophizing: rumination (exemplified by “I can't stop thinking about how much it hurts”), magnification (exemplified by “I worry that something serious may happen”), and helplessness (exemplified by “It's awful and I feel that it overwhelms me”). Higher scores indicate a higher level of catastrophizing. A total score above 30 is considered clinically relevant.

#### 2.4.6. Complications

Surgical reports as well as medical records after surgery were reviewed for the presence of complications such as seroma, wound infection, hematoma, and lymphedema.

### 2.5. Statistical analysis

The goal of the study was to describe the natural history of pain and sensory symptoms in patients with newly diagnosed cutaneous MM undergoing SLNB, with or without secondary CLND, or CLND-only, for 6 months after surgery. The primary outcome was overall pain within past 48 hours. Given the number of outcomes in the anticipated sample size, types of procedures, and variety of body locations, the analyses were planned to be primarily descriptive.

Where appropriate, a 2-sample test of 2 proportions was performed using STATA version 13.1 (STATA Corp, College Road, TX). *P* values <0.05 were considered statistically significant.

## 3. Results

### 3.1. Patients

**Figure 2.**
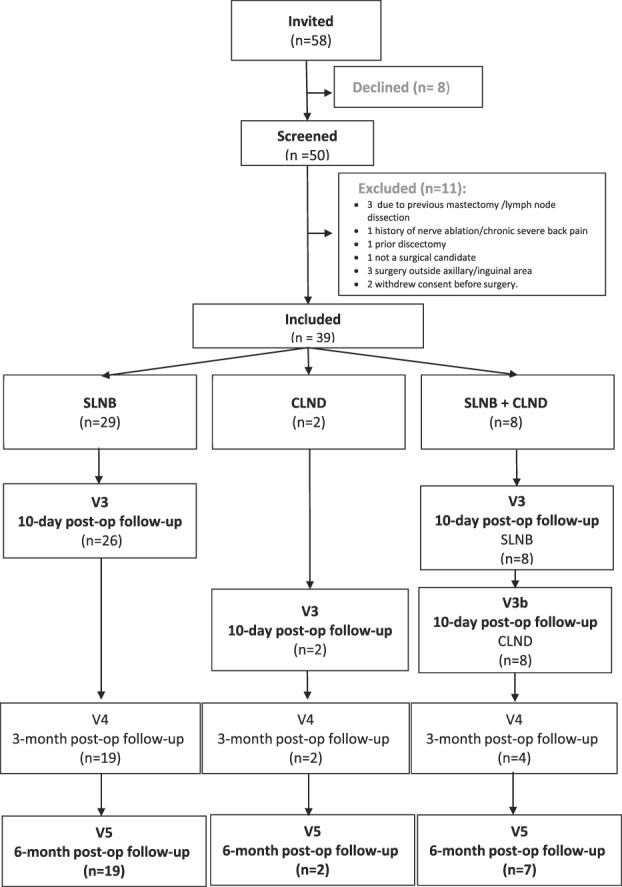
Participant flow through the study. CLND, complete lymph node dissection; SLNB, sentinel lymph node biopsy.

Patient flow is shown in Figure 2. A total of 58 patients were invited to participate, with 39 eligible and agreeing to participate. Of the 19 invited patients who did not participate, 8 declined, 3 were excluded due to previous mastectomy and lymph node dissection, 1 had a history of nerve ablation and chronic severe back pain, 1 had a prior discectomy, 1 was not a surgical candidate, 3 had surgery performed outside the axilla or inguinal regions, and 2 withdrew consent before surgery.

Twenty-nine underwent only SLNB, 2 underwent CLND without preceding SLNB, and 8 underwent SLNB followed by CLND (Fig. 2). Demographics of the 39 patients making up the study cohort are shown in Table 2.

**Table 2:**
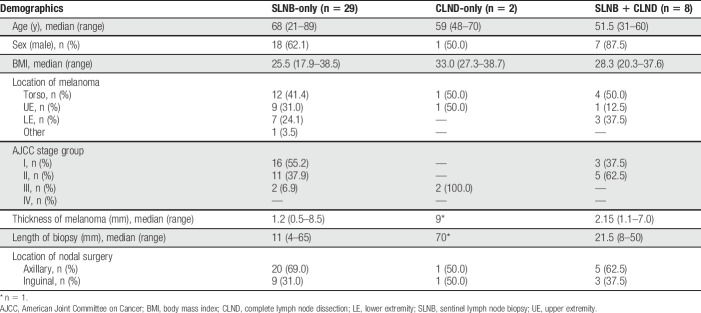
Patient demographics.

### 3.2. Follow-up

A total of 28 patients completed the full 6 months of follow-up; 19 of 29 patients undergoing SLNB-only, 2 of 2 undergoing CLND-only, and 7 of 8 undergoing SLNB-followed by CLND. Reasons for noncompletion were varied: 1 underwent CLND outside of the axillary or inguinal region, 2 withdrew consent after surgery due to the time requirements of study participation, 3 were lost to follow-up between the baseline visit and the 10-day postsurgery visit, and 5 were lost to follow-up before the 3-month and 6-month follow-up visits.

The 10-day postoperative visit with the surgeon was mandatory. More than half of the patients could only complete the 6-month follow-up evaluation through a telephone interview (usually because of distance and/or because surgical follow-up was not required at this time).

### 3.3. Pain

#### 3.3.1. Pain and analgesic use after surgery

**Figure 3.**
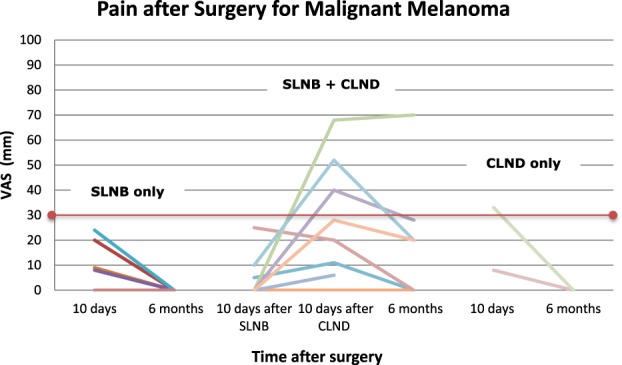
Pain intensity (VAS) in the group undergoing SLNB-only, SLNB followed by CLND, and CLND-only. The threshold for clinically meaningful pain (VAS ≥30 mm) is indicated. Data not shown for those patients who did not attend visit 3/visit 3b (10 days after surgery). CLND, complete lymph node dissection; SLNB, sentinel lymph node biopsy; VAS, Visual Analogue Scale.

At baseline (V1), no patients reported pain in the area where surgery was going to be performed. Figure 3 shows the pain intensity ratings for SLNB-only, SLNB + CLND, and CLND-only patients at 10 days and 6 months after surgery.

At 10 days after the SLNB procedure (n = 34, including the 8 who later underwent CLND), 12 of 34 SLNB patients reported pain (in 1 patient clinically meaningful at 89 mm) with a mean VAS of 7.4 mm (0–89). Sixteen of the 34 patients were still taking opioid analgesics. At 10 days after CLND, 9 of 10 patients reported pain (in 4 patients clinically meaningful pain) with a mean VAS of 26.6 mm (0–68). Seven of the 10 patients were still taking opioid analgesics. The difference in proportions with pain and clinically meaningful pain was significantly greater after CLND (pain *P* < 0.003; clinically meaningful pain *P* < 0.001).

At the 6-month visit, 0 of 19 SLNB patients, 4 of 7 SLNB + CLND patients (1 clinically meaningful at 70 mm, mean VAS 19.7 mm [0–70]), and 0 of 2 CLND-only patients reported pain [proportion with pain SLNB + CLND vs SLNB-only (*P* = 0.15) and vs CLND-only (*P* < 0.002)]. No patient reported use of any type of analgesic for SLNB/CLND symptoms at 6 months.

### 3.4. Sensory and pain symptoms

**Figure 4.**
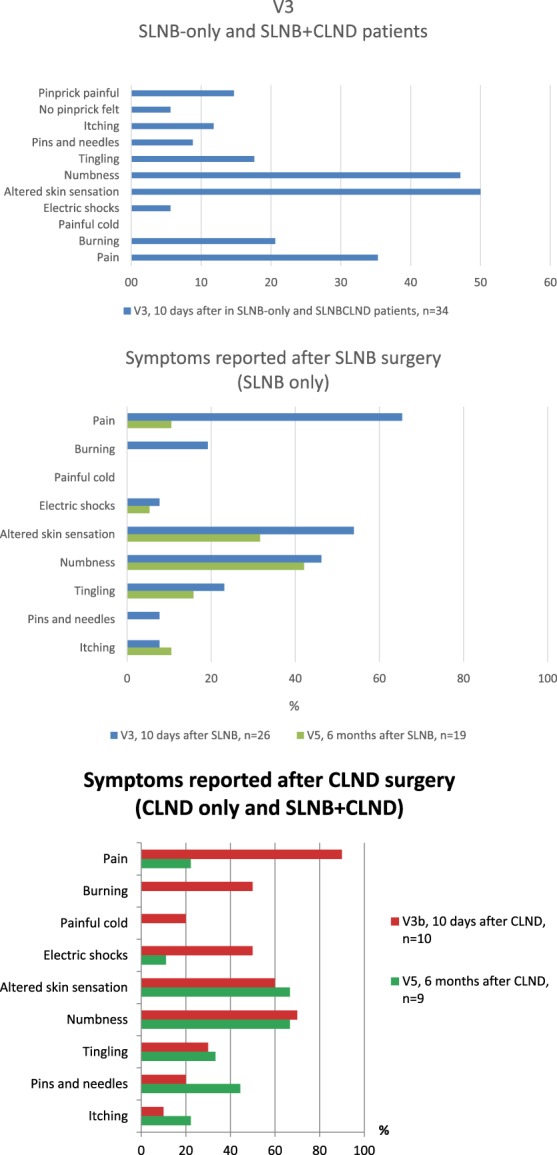
Percent of patients reporting neuropathic symptoms at SLNB (upper panel) or CLND (lower panel). CLND, complete lymph node dissection; SLNB, sentinel lymph node biopsy.

Figure 4 provides a bar graph for each symptom on the questionnaire in the SLNB-only and CLND patient groups. After the SLNB procedure (including the 8 who later underwent CLND), the most commonly reported sensory disturbances at the 10-day postsurgery visit were altered skin sensation in 53% (18/34 patients) and numbness in 46% (15/34 patients). At 6 months, symptoms of altered sensation or numbness were reported by 32% and 42% of SLNB-only patients. No patient reported burning, painful cold, or pins and needles sensations. Some patients checked “pain,” despite providing a 0/100 rating on the pain VAS.

The 10 patients undergoing CLND also reported altered skin sensation and numbness as the most common sensory disturbances at 10 days after surgery; 60% reported altered skin sensation and 70% reported numbness. At 6 months after the CLND, 67% and 67% of the 9 patients with data reported altered skin sensation or numbness. Compared to the SLNB-only group, the proportion reporting altered skin sensation was not significantly different (*P* = 0.08); the same was true for the proportion reporting numbness (*P* = 0.2). At 6 months, no patient reported burning or painful cold. However, as shown in Figure 4, the incidence of tingling, pins and needles, and itching was actually higher at 6 months.

### 3.5. Sensory examination

At 10 days, patients were difficult to examine reliably due to wound healing and bandaging issues at the site of interest that only 6-month data are reported. A total of 10 patients completed the sensory examination at 6 months after surgery. Of these, 7 were SLNB-only patients, 2 were SLNB + CLND patients, and 1 was a CLND-only patient.

At the visit 6 months after surgery, 0 of 7 SLNB-only patients had allodynia. All SLNB patients felt the Von Frey hair in the area of surgery at the visit 6 months after surgery. None of the SLNB-only patients reported pain with Von Frey hair stimulation.

For the 2 patients who underwent SLNB + CLND, 1 reported allodynia at the visit 6 months after CLND surgery. One of 2 patients felt the Von Frey hair at the visit 6 months after CLND surgery. No patients reported pain with Von Frey hair stimulation.

The patient undergoing CLND-only did not have allodynia at 6 months, was able to feel the Von Frey hair, and did not report it to be painful.

### 3.6. Questionnaires

At baseline, only a few patients had catastrophizing on the PCS (score ≥30), and abnormal anxiety or abnormal depression on the HADS (Table 3). These patients all reported several of the neuropathic symptoms; nevertheless, we found no clear pattern of more neuropathic symptoms in these catastrophizers nor a different combination of neuropathic symptoms from that of noncatastrophizers. There was no obvious change in the number of patients with catastrophizing, anxiety, or depression at the 6-month follow-up.

**Table 3:**
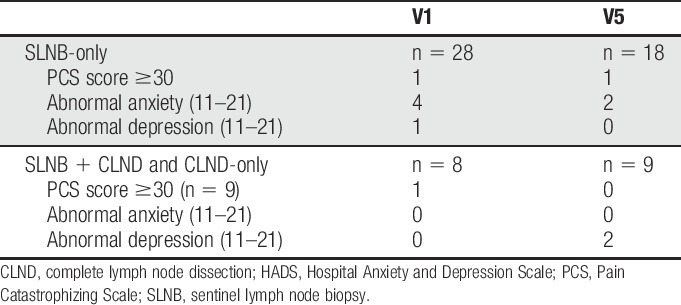
Pain Catastrophizing Scale scores ≥30 and HADS scores at visit 1 and visit 5.

### 3.7. Other complications

There were no severe complications during the study period. Transient minor complications included seroma, lymphedema, and cellulitis. None of the patients reported weakness, cognitive impairment, or issues with coordination at any visit.

## 4. Discussion

In a prospective cohort of 39 patients undergoing either SLNB or CLND for MM, significantly fewer patients had pain after SLNB than after CLND at 10 days after surgery. This result should be interpreted with some prudence due to the low number of patients. None of the SLNB-only patients reported pain at 6 months after surgery. All the patients with pain at 6 months after CLND had undergone the SLNB procedure before CLND.

The incidence of chronic postoperative pain varies across studies. This study is the first prospectively gathered quantitative data on pain severity in MM patients. These prospectively observed incidences of pain at 6 months are lower for SLNB but higher for CLND than in the retrospectively collected data of Høimyr et al., who reported “any chronic pain” in 14% of those undergoing SLNB-only (3% with clinically meaningful pain) and in 34% of those undergoing CLND (12% with clinically meaningful pain).^9^ After melanoma surgery, Kretschmer et al.^12^ reported long-term pain and nerve dysfunction in 1% after SLNB and 9% after CLND, but did not specify how pain was defined and did not distinguish between nerve dysfunction and pain. Reporting both the incidence of any pain and clinically meaningful pain helps define procedure-associated morbidity, and provides an estimate of how many patients might need ongoing treatment for chronic pain. A morbidity of 10% to 30% as measured by using “any pain” and 1% to 5% patients with clinically meaningful pain is in line with other conditions where pain is clearly defined.^10^ In axillary CLND, the intercostobrachial nerve is sacrificed during dissection, resulting in numbness of the upper inner arm. In inguinofemoral CLND, the anterior cutaneous branch of the femoral nerve is sacrificed, resulting in numbness of the anterior thigh. The higher incidence of any pain and clinically meaningful pain at 6 months after CLND in this study may be due to the more extensive nature of CLND (especially in patients who underwent SLNB a few weeks earlier) and associated lesioning of the nerves as part of the procedure.

Several questionnaires have been developed to identify neuropathic pain. The *Douleur Neuropathique en 4 Questions* (DN4) has shown a high specificity (90%) and sensitivity (83%); it has been validated in painful diabetic neuropathy, spinal cord injury, breast cancer surgery, cancer, and lower back pain.^4,5^ Neuropathic pain often includes spontaneous and evoked pain. Evoked pain manifests as allodynia and/or hyperalgesia on examination, and described as a burning sensation, painful cold, or electric shocks. Spontaneous pain, may be described as an unpleasant or abnormal sensation, called dysaesthesia (burning, tingling, itching, or pins and needles sensation). Therefore, in our study, we interviewed patients for the presence of descriptors from the DN4 for allodynia, hyperalgesia, and sensibility changes. The most common sensory disturbances reported were altered skin and numbness in the area of surgery. These symptoms were still present at 6 months in over 30% for those undergoing only SLNB and in 67% at 6 months in those undergoing CLND (with or without prior SLNB). The incidence of tingling, pins and needles, and itching was actually higher at 6 months in the CLND group than at ten days. These observed incidences are similar to the retrospective study of Høimyr et al.,^9^ where 32% of those undergoing SLNB-only and 82% of those undergoing CLND reported sensory disturbances. Similar findings have been observed in breast cancer patients undergoing the same nodal procedures.^1^

Recruiting a substantial number of patients was not difficult, with 39 successfully enrolled over 7 months from a single melanoma surgical center-of-excellence. The major weakness of this study is the low number of patients and large amount of missing data due to the small number of participants returning for physical examination at the later follow-up time points. For example, sensory testing data at 6 months after surgery were available in only 10 patients. Many patients were lost to follow-up or provided 6-month data only by phone. The CPMC, part of the Sutter Health system, is an urban teaching hospital with a large melanoma program. It draws Sutter Health patients from all of Northern California and has many outside referrals. A substantial proportion of patients travel long distances to San Francisco. At 6 months, SLNB-only patients are considered “cured” and may not be engaged with ongoing treatment. Complete lymph node dissection patients receiving chemotherapy from their local oncologist, or those engaged in a clinical trial, may have no further contact with the surgeon and would have little motivation to make the long trek back to the study site. Telephone follow-up is suboptimal compared with an in-person interview for obtaining a pain VAS and completing questionnaires, and precludes sensory testing.

Combined with the other advantages of the melanoma population (few comorbidities, adjuvant therapy only in advanced stages, and primary melanoma lesion outside of the SLNB and CLND surgical site) makes the postoperative pain after surgery for MM a viable model for studying neuropathic pain in a clinical setting. The strengths of this study are the prospective follow-up, standardized surgery performed by only one surgeon, and the interview-based symptom assessment.

An instructive prospective natural history study has been conducted in herpes zoster (HZ) patients systematically followed from rash onset with pain and sensory assessments, the capsaicin response test, and serial skin biopsy.^14^ At 3.9 years, none of the 29 subjects who had been pain-free at 6 months had a recurrence of pain. Only 2 of the 14 subjects with postherpetic neuralgia (PHN) at 6 months still had pain at 3.9 years. One subject with PHN at 6 months was free of symptoms at 3.9 years but had very mild pain at 7.7 years. Sensory function continued on a path toward normalization, but was still abnormal in many subjects, especially those who met criteria for PHN at 6 months. Even at 7.7 years, reinnervation of HZ-affected skin was not apparent. That study demonstrated that resolution of pain and resolution of sensory disturbances are separate processes; pain may fully resolve, although sensory disturbances and loss of cutaneous innervation are permanent.

As proposed by Kehlet and Rathmell,^11^ future studies would benefit from strategies that would make it easier for patients to be evaluated directly at later time points for better phenotyping, and from preoperative collection of blood samples for genetic testing. Established normal values as reference for training of investigators, intensive standardized sensory testing, regular in-person follow-up visits, and tracking more closely the analgesic use of patients are all elements that if included in standardized protocols would increase generalizability of the results and help identify small differences, and that otherwise could have been hidden in confounding from variability in patient management.

## 5. Conclusions

We conclude that chronic pain after melanoma surgery may depend on the type of surgery. The less invasive procedure of SLNB is rarely followed by long-term pain. Persistent pain and sensory symptoms appear to be more common in patients undergoing CLND (especially when preceded by SLNB), which should be taken into account when discussing treatment with the patients.

Prospectively studying neuropathic pain after melanoma surgery in a clinical setting may give valuable information on pain mechanisms and the process of acute pain becoming chronic, particularly because of the ease of patient recruitment, absence of preexisting pain, and the lack of complicating treatment comorbidities.

## Disclosures

The authors have no conflict of interest to declare.

## References

[1] Andersen K Kehlet H Persistent pain after breast cancer treatment: a critical review of risk factors and strategies for prevention. J Pain 2011;12:725–46. 21435953 10.1016/j.jpain.2010.12.005

[2] Balch CM Gershenwald JE Soong S Thompson JF Atkins MB Byrd DR Buzaid AC Cochran AJ Coit DG Ding S Eggermont AM Flaherty KT Gimotty PA Kirkwood JM McMasters KM Mihm MC Jr Morton DL Ross MI Sober AJ Sondak VK Final version of 2009 AJCC melanoma staging and classification. J Clin Oncol 2009;27:6199–206. 19917835 10.1200/JCO.2009.23.4799 PMC2793035

[3] Beitsch P Balch C Operative morbidity and risk factor assessment in melanoma patients undergoing inguinal lymph node dissection. Am J Surg 1992;164:462–6. 1443370 10.1016/s0002-9610(05)81181-x

[4] Bouhassira D Attal N Alchaar H Boureau F Brochet B Bruxelle J Cunin G Fermanian J Ginies P Grun-Overdyking A Jafari-Schluep H Lantéri-Minet M Laurent B Mick G Serrie A Valade D Vicaut E Comparison of pain syndromes associated with nervous or somatic lesions and development of a new neuropathic pain diagnostic questionnaire (DN4). PAIN 2005;114:29–36. 15733628 10.1016/j.pain.2004.12.010

[5] Bouhassira D Lantéri-Minet M Attal N Laurent B Touboul C Prevalence of chronic pain with neuropathic characteristics in the general population. PAIN 2008;136:380–7. 17888574 10.1016/j.pain.2007.08.013

[6] Gershenwald JE Scolyer RA Hess KR Sondak VK Long GV Ross MI Lazar AJ Faries MB Kirkwood JM McArthur GA Haydu LE Eggermont AMM Flaherty KT Balch CM Thompson JF; for members of the American Joint Committee on Cancer Melanoma Expert Panel and the International Melanoma Database and Discovery Platform. Melanoma staging: evidence based changes in the American Joint Committee on Cancer eighth edition cancer staging manual. CA Cancer J Clin 2017;67:472–92. 29028110 10.3322/caac.21409 PMC5978683

[7] Hettiaratchy S Dheansa B Powell B Lymphatic mapping and sentinel lymph node biopsy in patients with melanoma of the lower extremity. Plast Reconstr Surg 2000;106:734. 10.1097/00006534-200009030-00040 10987489

[8] Hettiaratchy SP Kang N O'Toole GA Powell BWEM Allan R Cook MG Sentinel lymph node biopsy in malignant melanoma: a series of 100 consecutive patients. Br J Plast Surg 2000;53:559–62. 11000070 10.1054/bjps.2000.3409

[9] Høimyr H Rokkones KA von Sperling ML Finnerup K Jensen TS Finnerup NB Persistent pain after lymph node excision in patients with malignant melanoma is neuropathic. PAIN 2011;152:2721–8. 21871733 10.1016/j.pain.2011.07.009

[10] Kehlet H Jensen TS Woolf CJ Persistent postsurgical pain: risk factors and prevention. Lancet 2006;367:1618–25. 16698416 10.1016/S0140-6736(06)68700-X

[11] Kehlet H Rathmell JP Persistent postsurgical pain: the path forward through better design of clinical studies. Anesthesiology 2010;112:514. 20124977 10.1097/ALN.0b013e3181cf423d

[12] Kretschmer L Thoms KM Peeters S Haenssle H Bertsch HP Emmert S Postoperative morbidity of lymph node excision for cutaneous melanoma-sentinel lymphonodectomy versus complete regional lymph node dissection. Melanoma Res 2008;18:16. 18227703 10.1097/CMR.0b013e3282f2017d

[13] Leong SPL Kim J Ross M Faries M Scoggins CR Metz WLR Cope FO Orahood RC A phase 2 study of (99m)Tc-tilmanocept in the detection of sentinel lymph nodes in melanoma and breast cancer. Ann Surg Oncol 2011;18:961–9. 21331809 10.1245/s10434-010-1524-z PMC3071527

[14] Reda H Greene K Rice FL Rowbotham MC Petersen KL Natural history of herpes zoster: late follow-up of 3.9 years (n = 43) and 7.7 years (n = 10). PAIN 2013;154:2227–33. 23719573 10.1016/j.pain.2013.04.015

[15] Slagelse C Petersen KL Dahl JB Finnerup K Greene K Leong SP Levine J Rowbotham M Werner MU Finnerup NB Persistent postoperative pain and sensory changes following lymph node excision in melanoma patients: a topical review. Melanoma Res 2014;24:93. 24346167 10.1097/CMR.0000000000000041

[16] Sullivan MJL Bishop SR Pivik J The Pain Catastrophizing Scale: development and validation. Psychol Assess 1995;7:524.

[17] Urist MM Maddox WA Kennedyy JE Balch CM Patient risk factors and surgical morbidity after regional lymphadenectomy in 204 melanoma patients. Cancer 1983;51:2152–6. 6839303 10.1002/1097-0142(19830601)51:11<2152::aid-cncr2820511134>3.0.co;2-7

[18] de Vries M Vonkeman WG van Ginkel RJ Hoekstra HJ Morbidity after axillary sentinel lymph node biopsy in patients with cutaneous melanoma. Eur J Surg Oncol 2005;31:778–83. 15993029 10.1016/j.ejso.2005.05.003

[19] Zigmond AS Snaith RP The Hospital Anxiety and Depression Scale. Acta Psychiatr Scand 1983;67:361–70. 6880820 10.1111/j.1600-0447.1983.tb09716.x

